# Microstructural Stability and High-Temperature Mechanical Behavior of Al–Ni–Zr Alloy Strengthened by L1_2_-Al_3_Zr Precipitates

**DOI:** 10.3390/ma18133068

**Published:** 2025-06-27

**Authors:** Jan Šmalc, Adam Zaky, Boštjan Markoli, Roman Šturm

**Affiliations:** 1 Faculty of Mechanical Engineering, University of Ljubljana, Aškerčeva 6, 1000 Ljubljana, Slovenia; jan.smalc@fs.uni-lj.si; 2 Faculty of Natural Sciences and Engineering, University of Ljubljana, Aškerčeva 12, 1000 Ljubljana, Slovenia; adam.zaky@ntf.uni-lj.si (A.Z.); bostjan.markoli@ntf.uni-lj.si (B.M.)

**Keywords:** aluminum–nickel eutectic, precipitation strengthening, L1_2_-Al_3_Zr precipitates, microhardness, accelerated creep test

## Abstract

Aluminum alloys based on the eutectic Al–Ni system are a promising class of lightweight materials for applications at elevated temperatures owing to the thermal stability of the eutectic Al_3_Ni phase. In this study, the eutectic Al–Ni alloy was modified by the addition of 0.6 wt.% Zr to enhance the α_Al_ matrix by precipitation strengthening. The alloys were cast and subjected to T5 heat treatment followed by long-term isothermal aging at 350 °C. A comprehensive study was carried out to evaluate the evolution of microstructure, microhardness and mechanical performance over time. The formation of fine, coherent L1_2_-Al_3_Zr precipitates contributed to significant strengthening, as reflected by a ~60% increase in microhardness and an approximately twofold improvement in room temperature (RT) yield strength. A TEM analysis of the L1_2_-Al_3_Zr precipitates showed relatively good thermal stability after 30 days. Despite the improved mechanical properties at room temperature, the alloy did not retain this improvement when tested at 300 °C. Nevertheless, these results provide a comprehensive insight into the aging and thermal stability of Al–Ni–Zr alloys.

## 1. Introduction

In the search for a castable aluminum alloy that can withstand high temperatures (i.e., above 200–300 °C) [1], binary alloys from Al–Ni, Al–Ce and Al–La systems have attracted increasing research interest [2,3,4,5,6,7]. The Al–Ni binary system has a eutectic composition at 6.1 wt.% Ni, and this eutectic alloy exhibits desirable properties such as good castability and resistance to hot-tearing [8,9]. During solidification, the eutectic alloy from the Al–Ni system forms eutectic colonies consisting of the Al_3_Ni eutectic phase, which is characterized by a fibrous or rod-like morphology and is surrounded by an α_Al_ matrix. While the Al_3_Ni fibers exhibit good thermal and chemical stability at elevated temperatures [10,11], the α_Al_ matrix remains relatively soft due to the limited solubility of Ni in α_Al_. Therefore, additional alloying elements are required to increase the strength of the α_Al_ matrix.

Previous studies have shown that the addition of transition metals (TMs) and rare earths (REs) to dilute aluminum alloys promotes precipitate formation upon aging, which significantly improves the properties of the matrix [12,13]. These precipitates predominantly form intermetallic tri-aluminide phases with a general stoichiometry of Al_3_X (where X = Zr, Ti, Sc and Er) [14,15,16]. In particular, Al_3_Zr and Al_3_Ti precipitates initially form a metastable cubic L1_2_ structure, while Al_3_Sc and Al_3_Er directly form stable cubic L1_2_ structures [12]. The presence of these intermetallic phases contributes significantly to the high-temperature stability of the alloy [14,17], effectively inhibits recrystallization [18,19] and improves the creep resistance of aluminum alloys [20,21]. Small additions of alloying elements such as Zr and Sc to the eutectic Al–Ni system provide a viable means of introducing additional precipitation hardening to complement the inherent benefits of the eutectic microstructure. Previous studies on Sc and Zr additions in the Al–Ni system indicate positive effects on mechanical properties, including increased microhardness at RT and improved creep resistance [22,23].

This study investigates the feasibility of the widespread production of a castable Al–Ni–Zr alloy that exhibits increased thermal stability above 250 °C. Such an alloy would be well suited to high-temperature applications in the automotive, aerospace and power generation industries, including components such as aluminum pistons and water-jacketed turbine housings. To achieve the desired alloy composition, non-equilibrium solidification conditions must be maintained, as higher cooling rates promote the increased supersaturation of Zr within the α_Al_ matrix [24]. After solidification and subsequent heat treatment (T5), the formation of Zr-rich precipitates occurs, as confirmed by previous studies [23]. Based on the results of our previous research [25], an aging temperature of 350 °C was chosen to optimize the precipitation process and thus maximize the precipitate strength throughout the microstructure without negatively affecting the integrity of the eutectic phases. The objective of this study was to investigate the influence of small Zr addition to the eutectic Al–Ni alloy system and the subsequent aging treatment at 350 °C. The main focus was on characterizing the microstructural evolution and evaluating the changes in microhardness during the aging. In addition, tensile tests and accelerated compressive creep tests were performed to quantitatively evaluate the strengthening effects associated with the precipitation of Al_3_Zr precipitates.

## 2. Materials and Methods

The experimental alloys were produced using an electric resistance furnace from technically pure aluminum (99.7%; Mozal SA, Boane, Mozambique), high-purity nickel (99.99%; F. Bauerstatter GES.M.B.H, Vienna, Austria) and an Al–10 wt.% Zr master alloy (KBM Affilips, Oss, The Netherlands). Two alloy compositions were investigated: Al–6.1Ni (referred to as AN) and Al–6.1Ni–0.6Zr (referred to as ANZ). The materials were melted in a graphite crucible and held at 850 °C for at least 30 min after the addition of the Al–Zr master alloy to ensure the complete dissolution of the alloying elements. Before casting, the melt was mechanically stirred and the oxide film was removed from the surface. The prepared melt was then poured into a copper mold, resulting in a test specimen with a dimension of 24 mm × 12 mm × 130 mm.

The casting alloys were aged in air at 350 °C in a muffle furnace EUP-K 20/1200 (Bosio, Celje, Slovenia) and then immediately quenched in water to ambient temperature. The designation of heat-treated samples follows the format Alloy-x, where x indicates the duration of aging at 350 °C in hours. For example, the designation ANZ-12 refers to the Al–Ni–Zr alloy that has been aged at 350 °C for 12 h. The samples for microstructural characterization by light optical microscopy (LOM) and scanning electron microscopy (SEM) were first ground with emery paper with increasing grain sizes of 600, 800, 1000 and finally 1200. The samples were then polished with 3 µm diamond paste, followed by a final polish with 1 µm diamond paste. The LOM analyses were carried out using Axioscope 5 (Carl Zeiss GmbH, Oberkochen, Germany). The chemical composition of the alloys was determined by energy dispersive X-ray spectroscopy with Ultim Max-EDS (Oxford Instruments, Oxford, UK) using a Quanta 650 (Thermo Fisher Scientific, Waltham, MA, USA) SEM operated at an accelerating voltage of 20 kV over an analyzed area of 2.5 mm × 2.5 mm. At least three measurements were performed per sample at randomly selected positions. An additional analysis of the phase fraction was carried out using SEM micrographs and the open-source software Fiji (ImageJ-version 1.54p). The characterization of the precipitates was carried out using a JEM-2100 transmission electron microscope—TEM (JEOL, Tokyo, Japan)—operated at an accelerating voltage of 200 kV. The TEM samples were first mechanically ground to a thickness of approximately 80 µm and then dimpled to a thickness between 20 and 30 µm. The final thinning was carried out using a precision ion polishing system—PIPS model 691 (Gatan, Ametek, Pleasanton, CA, USA)—with argon ions, starting with an ion energy of 4.5 keV and ending with a low-energy polishing step at 2 keV.

Vickers microhardness measurements were carried out using a Wilson VH 1202 (Buehler, Bühler, Switzerland) hardness tester under the HV 0.1 load scale, with at least ten individual measurements per sample. Electrical conductivity was assessed with a Hocking Phasec 2s (General Electric, Evendale, OH, USA) with a 960 kHz probe, with a minimum of four measurements per sample. The tensile tests were performed using a Z250 (Zwick Roell, Ulm, Germany) universal testing machine equipped with a 50 kN load cell and a HTO-08 (Maytec, Singen, Germany) heating furnace. The tensile specimens were machined and heat-treated according to ASTM E8M-04 [26] to achieve the desired condition. Cylindrical specimens with a diameter of 5 mm and a length of 10 mm were prepared for accelerated compression creep tests. These creep tests were performed with a DIL 805A/D (Ta Instruments, New Castle, DE, USA) dilatometer, applying a constant compressive force at predefined temperatures. The applied forces were selected based on previous compression tests at corresponding temperatures so that the applied stresses were between 0.4 and 0.6 of the Rp_0.01_ value for both alloy types. Accelerated creep tests were carried out at temperatures of 250, 275 and 300 °C on samples that had previously been heat-treated.

## 3. Results and Discussion

### 3.1. Chemical Composition

The chemical composition and designation of the alloys produced are provided in Table 1. The chemical compositions were determined using an SEM with an EDS detector. The traces of Fe detected in the alloys (Table 1) originated as impurities from technically pure aluminum and the Al–Zr master alloy.

### 3.2. Microhardness and Electrical Conductivity

**Figure 1:**
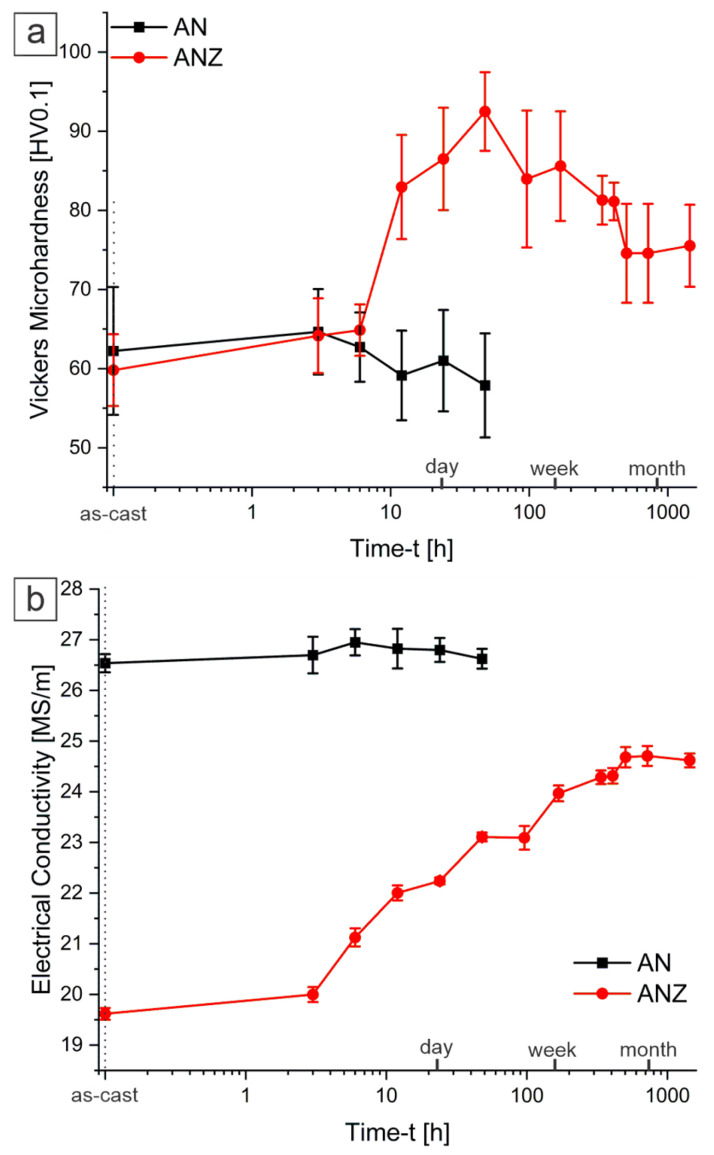
Evolution of Vickers microhardness (**a**) and electrical conductivity (**b**) after aging at 350 °C for alloys AN and ANZ.

Figure 1 shows the evolution of microhardness and electrical conductivity for the AN and ANZ alloys during aging at 350 °C. As can be seen in Figure 1a, the AN alloy shows a minimal change in microhardness aging from an initial value of 62 HV0.1 to approximately 58 HV0.1. Comparable values for the as-cast microhardness of the Al-6Ni alloy were previously reported by Suwanpreecha et al. [22] and Michi et al. [27]. Furthermore, Suwanpreecha also reported unchanged microhardness after the exposure of Al-6Ni at 350 °C for a duration of two months. In contrast, the ANZ alloy shows considerable precipitation strengthening upon aging. The ANZ alloy initially has a lower microhardness than the AN alloy. Although Zr is added to the solid solution, the strengthening effect of the solid solution is less pronounced than the microstructural changes induced by the Zr addition. The microhardness behavior of an alloy with a comparable chemical composition has already been described in our earlier work [25]. During the initial 6 h of aging, a modest increase in microhardness is observed, followed by a significant increase after 12 h, indicating a pronounced age-hardening effect. Accordingly, the values of electrical conductivity closely follow the trend of microhardness. The peak value of microhardness is reached after about 48 h of aging with 92 HV0.1, an increase that can be attributed to the formation of fine, coherent Zr-containing precipitates. In comparison, Belov et al. [28] and Michi et al. [27] reported peak-aged microhardness (780 and 850 MPa), although the aging was carried out at a higher temperature (400 °C) and shorter time (24 h and 20 h), respectively.

Continued aging from 48 to 504 h leads to a gradual decrease in microhardness to around 75 HV0.1, which is due to the coarsening of the precipitates and the associated depletion of Zr from the α_Al_ matrix. During this aging interval, the electrical conductivity increases steadily. It is noteworthy that the microhardness stabilizes at around 75 HV0.1 after 504 h, indicating a balance between the precipitate coarsening of the precipitates and the depletion of Zr in the matrix. This stabilization is accompanied by relatively constant electrical conductivity.

### 3.3. Microstructure

#### 3.3.1. Optical Microscopy (LOM)

**Figure 2:**
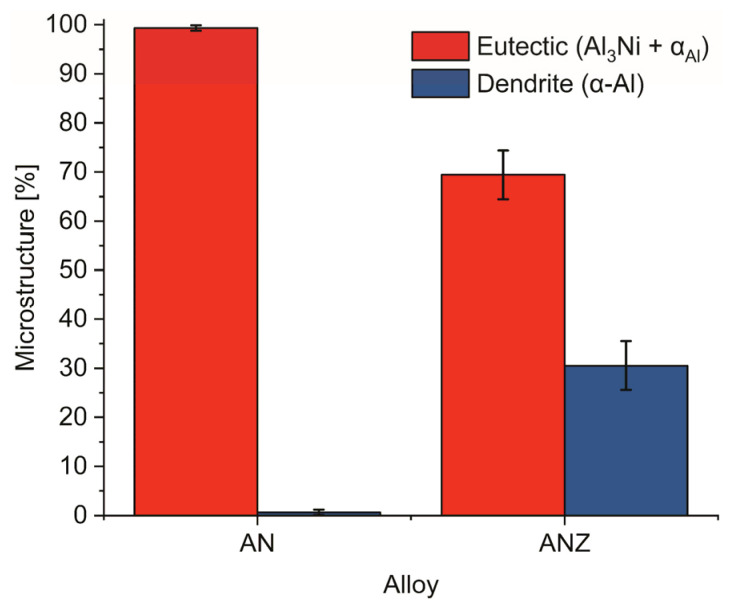
Microstructure fraction for alloys AN and ANZ.

**Figure 3:**
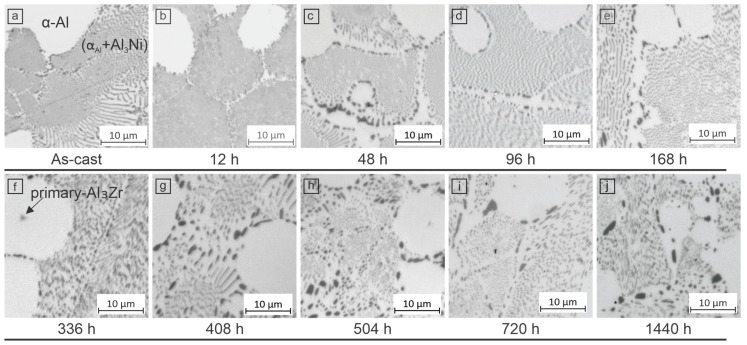
Optical micrographs of microstructural evolution of alloy ANZ after aging at 350 °C: (**a**) As cast; (**b**) 12 h; (**c**) 48 h; (**d**) 96 h; (**e**) 168 h; (**f**) 336 h; (**g**) 408 h; (**h**) 504 h; (**i**) 720 h; and (**j**) 1440 h.

The changes in microstructural constituents are presented in Figure 2, where the alloy AN exhibits a predominantly eutectic microstructure. However, the introduction of Zr causes an increase in α_Al_ dendrites, which represent ~30% of the microstructure, which was also reported in our previous work [25]. The equiaxed morphology of the α_Al_ dendrites is attributed to the addition of Zr, which promotes solidification through a peritectic reaction with primary Al_3_Zr phases, as shown in Figure 3f. The addition of 0.6 wt.% Zr promotes the nucleation of α_Al_ via heterogeneous nucleation initiated by primary Al_3_Zr particles.

The eutectic Al_3_Ni phase mainly exhibits a fibrous morphology, which is embedded in the eutectic α_Al_ phase and jointly forms eutectic colonies. However, a partial transition from the fibrous to the lamellar morphology of Al_3_Ni can be observed, especially in the vicinity of α_Al_ dendrites formed during solidification. The relatively pure composition of the dendrites (due to the low solubility of nickel and iron in aluminum) causes impurity atoms to be pushed towards the solidification front. This process inhibits the formation of a fibrous eutectic and promotes the lamellar morphology of Al_3_Ni, and this has previously also been observed by Kakitani et al. [29]. Furthermore, the distribution of these eutectic morphologies indicates an influence of latent heat release during solidification. Chemical analyses also indicate a higher iron concentration within the lamellar Al_3_Ni phases, which underlines a preferential affinity for Fe (confirmed by SEM).

Aging the ANZ alloy at 350 °C for 12 h shows no significant microstructural changes (Figure 3b). Slight microstructural changes can be observed, with the microhardness of the alloy peaking after 48 h at 350 °C. There is some coarsening of the Al_3_Ni phase at the edges of the colonies with a focus on the lamellar phase, while aging has little effect on the morphology of the Al_3_Ni fibers. After 168 h (Figure 3e), darker intermetallic phases, probably rich in Fe and/or Ni, form at the colony boundaries and the dendritic edges. The lamellae start to coarsen faster, while the fibrous eutectic shows only minimal coarsening, mainly at the edges of the colonies. After further aging for 408 and 504 h (Figure 3g,h), the lamellar eutectic phases show pronounced segregation and growth, while the fibrous eutectic phases undergo moderate coarsening. At the same time, intermetallic precipitates increasingly accumulate at the colony boundaries. After 720 h of aging, the microstructure approaches a state of relative stability, although the lamellar phases continue to show gradual coarsening and agglomeration into larger intermetallic areas. After prolonged aging (1440 h at 350 °C), the eutectic colony boundaries continue to expand. It is noticeable that the morphology of the dendritic α_Al_ phases changes from globular to spheroidal shape. This development can be attributed to the continued segregation of the elements, especially in regions that were originally dominated by lamellar eutectic phases. The increasing segregation causes these regions to resemble dendritic α_Al_ structures, accompanied by a large, randomly distributed, dark intermetallic Al_3_Ni phase (Figure 3j).

#### 3.3.2. Scanning Electron Microscopy (SEM)

**Figure 4:**
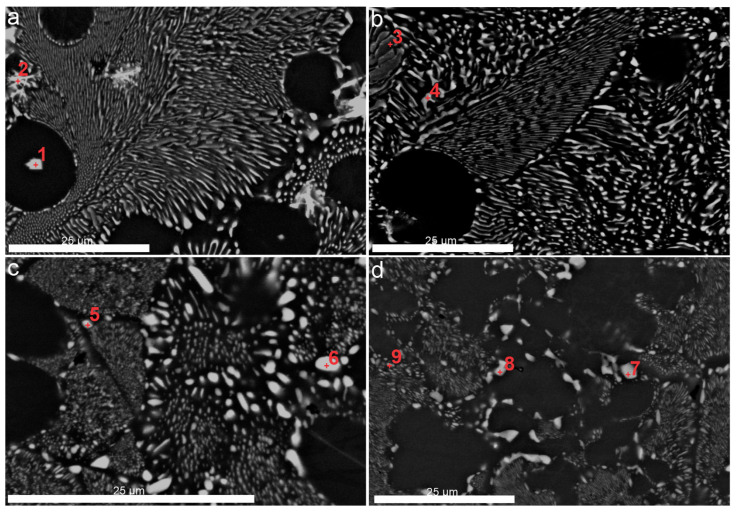
SEM micrographs with EDS analyses (marked red spot): (**a**) Sample ANZ-48; (**b**) Sample ANZ-168; (**c**) Sample ANZ-720; and (**d**) Sample ANZ-1440.

Figure 4 shows SEM images with the corresponding EDS analyses summarized in Table 2. Figure 4a highlights the specific regions where EDS measurements were performed on the primary Al_3_Zr phase, which appears in two different morphologies. As reported by Khvan et al. [30], the morphology labeled by spot 1 is referred to as a “porous crystal” or “flower crystal”. In addition, the second, plate-like morphology corresponding to spot 2 was observed by Michi et al. [27]. According to the SEM-EDS results presented in Table 2, the chemical compositions at spots 1 and 2 are largely similar, with the exception of a higher Ni content in one of the regions, which is most likely due to an analytical deviation. Spots 3 and 4 reflect the chemical composition of the eutectic lamellae. It is noteworthy that the morphology at spot 4 is slightly altered, indicating the instability of the lamellar eutectic phase after one week of aging in the ANZ alloy. The microstructures shown in Figure 4c,d exhibit significant changes, in particular the agglomeration of phases, predominantly at the peripheries of the colonies. In Figure 4c, the lamellar eutectic (α_Al_ + Al_3_Ni) morphology appears disrupted and coarsened into larger, irregular phases. The chemical composition of 5–9 spots indicates coarsened regions of the Al_3_Ni phase, with a slight enrichment of Fe, a feature more commonly observed at colony boundaries. The coarsening of the Al_3_Ni phase has already been documented by Czerwinski [10].

#### 3.3.3. Transmission Electron Microscopy (TEM)

**Figure 5:**
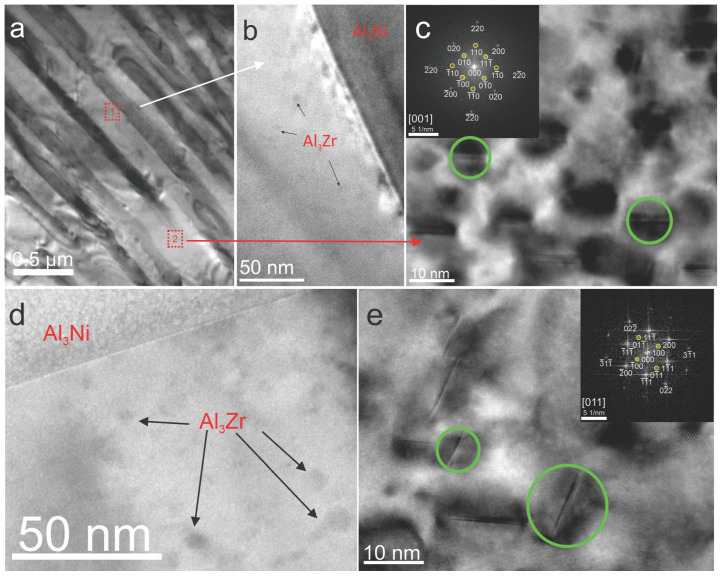
The TEM analysis of the ANZ alloy aged at 350 °C; (**a**) a bright field image of the ANZ-48 sample and (**b**) magnification of the area marked with the dashed square 1, in which the Al_3_Zr precipitates marked with arrows can be seen; (**c**) a bright field image of the ANZ-48 sample with green circles indicating the L1_2_-Al_3_Zr precipitates and the FFT pattern in which yellow circles indicate the L1_2_-Al_3_Zr positions; (**d**) a bright field image of the ANZ-720 sample with arrows indicating the Al_3_Zr precipitates; and (**e**) a bright field image of the 720 h aged ANZ alloy with green circles indicating the L1_2_-Al_3_Zr precipitates and the FFT pattern of the corresponding diffraction in which yellow circles indicate the L1_2_ positions.

In order to characterize the precipitates on the nanoscale, a detailed TEM analysis was carried out. Figure 5a shows the eutectic microstructure of the alloy ANZ, which was aged at 350 °C for 48 h. Figure 5b,c show magnified images of the areas labeled “1” and “2”, respectively. Figure 5b clearly shows the presence of fine Al_3_Zr precipitates in close proximity to the eutectic Al_3_Ni fibers. Figure 5c also shows areas with L1_2_-Al_3_Zr precipitates, some of which are marked by green circles. Figure 5c shows the darker areas in which strain contrast between matrix and precipitates can be seen. Accordingly, the FFT diffraction pattern in Figure 5c shows the positions of the α_Al_ matrix and the coherent precipitates. The diffraction pattern indicates the positions (yellow circles) of the spots for the ordered f.c.c. L1_2_ unit cell of the Al_3_Zr precipitate. Figure 5d,e show the microstructure of the ANZ-720 alloy and confirm the presence of Al_3_Zr precipitates next to eutectic Al_3_Ni fibers, confirming that the effective precipitation hardening of the ANZ alloy has taken place. The formation of these fine precipitates during aging predominantly within the eutectic α_Al_ phase contributes significantly to the strengthening of the weaker component of the eutectic microstructure. Figure 5e and the FFT diffraction pattern also show the presence of cubic L1_2_-Al_3_Zr precipitates after 720 h at 350 °C.

**Figure 6:**
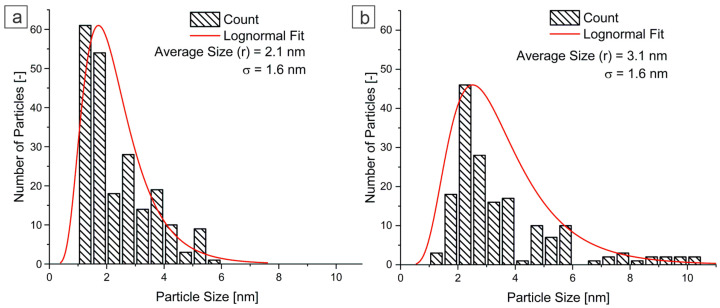
Histogram of average size of precipitates with lognormal distribution for samples: (**a**) ANZ-48; (**b**) ANZ-720.

Previous studies on Al-based alloys have reported comparable Al_3_Zr precipitate sizes under different conditions. For example, Michi et al. [27] observed Al_3_Zr precipitates with an average radius of 2.1 ± 0.4 nm after aging at 425 °C for 24 h. Pandey et al. [31] reported Al_3_Zr precipitates with an average radius of 2.5 ± 1.0 nm after 10 h and 4.0 ± 1.0 nm after 100 h of aging at 400 °C. In addition, Fan and Makhlouf [15] produced Al_3_(Zr_x_,V_1−x_) precipitates with an average radius of 2.6 nm after aging at 400 °C for 32 h. Knipling et al. [14] recorded Al_3_Zr precipitates with a radius of 5.1 ± 1.7 nm after aging at 425 °C for 400 h in dilute binary Al–Zr alloys. Figure 6 shows the lognormal distributions of precipitate sizes in ANZ alloy samples aged at 350 °C for 48 and 720 h (ANZ-48 and ANZ-720), respectively. The distribution of L1_2_-Al_3_Zr precipitates was assessed using size measurements from TEM images in brightfield and HRTEM modes. The TEM analysis was performed at magnifications from 100,000× to 500,000× and an accelerating voltage of 200 kV. Precipitate size measurements were performed using the open-source software Fiji, analyzing at least 170 precipitates per sample. As can be seen in Figure 6a,b, the average precipitate radius (r) of the L1_2_-Al_3_Zr phase fitted with a lognormal distribution is r = 2.1 ± 1.6 nm for ANZ-48 and r = 3.1 ± 1.6 nm for ANZ-720. With continued aging at a temperature of 350 °C, there is a tendency for small precipitates with higher curvature to dissolve, as can be seen from the changes in the distribution in Figure 6. When small precipitates dissolve, the resulting solute diffuses towards larger precipitates, causing them to grow and hence the total interfacial energy to decrease. Comparing the results obtained in this study with those of the previously cited references, there is good agreement. However, it is important to note that most of these studies were conducted at higher aging temperatures (400–425 °C). Consequently, the larger precipitate sizes are primarily attributed to the increased diffusion rate of Zr in aluminum at higher temperatures, where the diffusivity coefficient is about two orders of magnitude higher than at 350 °C. Furthermore, it is important to emphasize that although the average size of the L1_2_-Al_3_Zr radius increased at aging between 48 h and 720 h, the coherence of the precipitates is maintained.

### 3.4. Tensile Tests

**Figure 7:**
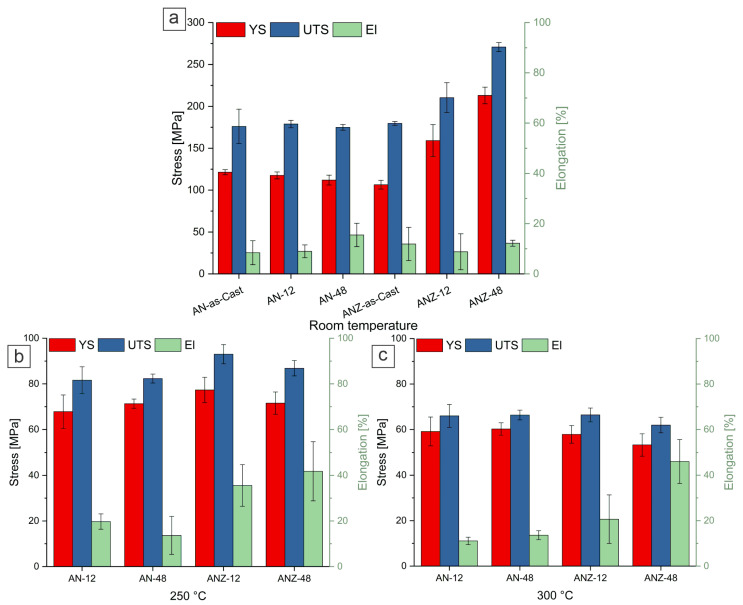
Results of tensile test analysis; (**a**) at room temperature (RT); (**b**) at 250 °C; and (**c**) at 300 °C.

Tensile test experiments were carried out at RT, 250 °C and 300 °C and the results are presented in Figure 7. Per each tensile test parameter, at least three specimens were measured. The tensile tests performed at room temperature did not reveal any significant differences between the eutectic AN alloy in terms of ultimate tensile strength (UTS) and yield strength (YS), even when exposed to 350 °C for up to 48 h; however, it showed a slight increase in elongation (El), indicating the good thermal stability of the eutectic Al_3_Ni fibers. The results for eutectic alloys are in good agreement with those previously reported by Koutsoukis et al. [32]. Similarly, the as-cast ANZ alloy showed similar mechanical properties to the eutectic AN alloy, apart from a slightly lower initial yield strength. In particular, the ANZ-12 alloy showed a 17% improvement in UTS and a 50% increase in YS compared to the as-cast alloy. The highest improvement in mechanical properties was observed with the ANZ-48 alloy, which achieved a remarkable 102% increase in yield strength and a 50.8% improvement in UTS compared to the as-cast alloy.

At an elevated test temperature of 250 °C, the tensile properties of the AN alloy remain similar under the tested conditions. However, the ANZ-48 alloy shows a slight improvement (Figure 7) in mechanical properties, while the ANZ-12 alloy shows a significant increase in YS and UTS. The most significant difference is seen in tensile elongation (El), with the ANZ series alloys exhibiting significantly higher elongation compared to their AN counterpart. Further tensile tests at 300 °C show a significant reduction in mechanical properties, which is particularly noticeable in the Zr-containing alloys. At this temperature, the UTS values of the AN-12, AN-48 and ANZ-12 alloys converge, while ANZ48 has a slightly lower UTS value, indicating the susceptibility of the Zr-strengthened alloys to softening at higher temperatures.

### 3.5. Accelerated Compressive Creep Test

**Figure 8:**
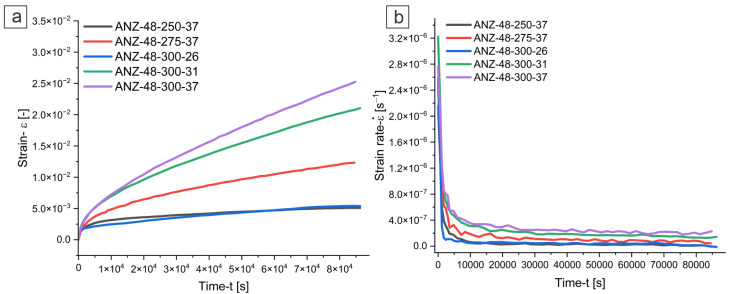
Compressive creep tests curves: strain–time (**a**) and strain rate–time (**b**).

Figure 8 shows the accelerated compression creep behavior of the investigated ANZ-48 alloy under different test conditions. All experiments shown in Figure 8a exhibit a monotonous creep response. Figure 8b shows the corresponding strain rate curves for the ANZ-48 alloy, where a pronounced primary creep phase is followed by a secondary (steady-state) creep phase. The average minimum creep rates for all investigated alloys were extracted from the secondary phase, where, from average minimum creep rates, further evaluations were carried out to determine the activation energy for creep (Q_c_) and the stress exponent (*n*), the results of which are summarized in Table 3.

It is evident from Table 3 that the activation energy for creep in the AN alloy is the lowest of the alloys analyzed and closely matches the activation energy for pipe diffusion (Q ≈ 82 kJ/mol) [33]. In addition, a stress exponent of n = 3 was determined for the AN alloy, indicating dislocation activity with a dislocation glide mechanism [34]. Taken together, these observations indicate that the dominant creep mechanism in the AN alloy under the applied test conditions is most likely controlled by a combination of pipe diffusion and limited viscous dislocation glide. This is not consistent with previous studies by Michi et al. [27] and Suwanpreecha et al. [22], who investigated eutectic Al–Ni alloys and reported higher stress exponents, suggesting the existence of threshold stress. However, it is important to point out that the creep stresses applied in the present study were lower than those used in previous studies. Table 3 shows that the Q_c_ for the ANZ-12 and ANZ-48 alloys is close the lattice self-diffusion energy of pure aluminum (Q ≈ 142 kJ/mol) [33]. This increase, together with the increased stress exponents, indicates a transition to a different creep regime. In particular, the combination of higher Q_c_ and stress exponent values indicates that the dominant creep mechanism is most likely self-diffusion-driven dislocation climbing, which is usually characterized by the power-law creep regime [35]. These results are in good agreement with the observations of Michi et al. [27], who found a detrimental effect on creep resistance associated with a low volume fraction of α_Al_ dendrites. The shift in the creep mechanism in the ANZ-12 and ANZ-48 alloys correlates with a slight reduction in their creep resistance. This behavior can be attributed to microstructural changes in these alloys, in particular an increased volume fraction of α_Al_ dendrites of up to about 30% (Figure 2), which can disrupt the efficiency of load transfer from the matrix to the Al_3_Ni fibers and redirect it towards the α_Al_ dendrites. The results indicate that the increased volume fraction of the α_Al_ dendrites in the microstructure can counteract the strengthening effect of the L1_2_-Al_3_Zr precipitates and thus reduce the overall creep resistance.

## 4. Conclusions

In this study, eutectic Al–Ni and Al–Ni–Zr cast alloys were investigated, focusing on the development of microstructure, microhardness and electrical conductivity during isothermal aging at 350 °C for a period of two months.

1. The introduction of 0.6 wt.% Zr into the eutectic Al–Ni system leads to an increased proportion of equiaxed α_Al_ dendrites (~30%). Aging alloy at 350 °C leads to a peak in microhardness at 92 HV0.1 after 48 h. Extended aging results in the partial coarsening of the microstructure, with the microhardness stabilizing at 75 HV0.1 after 500 h.
2. Upon aging, coherent L1_2_-Al_3_Zr precipitates with an average radius of r = 2.1 ± 1.6 nm were observed at peak aging conditions (350 °C, 48 h). Their high resistance to coarsening is evident after 30 days of aging, where the average radius of the precipitates increased only slightly to r = 3.1 ± 1.6 nm, while coherence with the matrix was maintained.
3. The tensile tests of the peak-aged ANZ-48 sample revealed a significant increase in yield strength from 106 MPa to 213 MPa at room temperature. However, this increase is not retained at elevated temperatures (300 °C), where the yield strength drops to 53 MPa. The YS value is in a similar range to the alloy without Zr addition.
4. Accelerated compressive creep tests indicated that the AN alloy exhibits a creep-dominated mechanism of pipe diffusion and limited viscous dislocation glide, whereas the ANZ alloy follows a power-law creep regime under the conditions tested.

## Figures and Tables

**Table 1:** SEM analysis of chemical composition and designation of alloys in wt.%.

Expected Composition	Ni	Zr	Fe	Al	Designation
Al-6.1Ni	6.41 ± 0.14	0.00	0.14 ± 0.02	bal.	AN
Al-6.1Ni-0.6Zr	6.28 ± 0.01	0.56 ± 0.1	0.11 ± 0.05	bal.	ANZ

**Table 2:** Results of SEM-EDS analyses in wt.% from Figure 4.

Spectrum Label	1	2	3	4	5	6	7	8	9
Al	77.4	75.9	91.1	84.0	82.9	68.7	72.3	73.5	83.2
Ni	0.8	7.2	8.5	15.5	15.3	31.2	27.5	26.3	16.6
Fe	-	-	0.1	0.2	1.8	0.1	0.2	0.2	0.2
Zr	21.6	16.4	0.3	0.3	-	-	-	-	-
Si	0.2	0.5	-	-	-	-	-	-	-

**Table 3:** Results of average minimal strain rates for each condition, activation creep energy and stress exponents of alloys AN, ANZ-12 and ANZ-48.

Temperature T [°C]	Stress σ [MPa]	$\mathrm{Strain}\;\mathrm{Rate}\;\dot{\varepsilon }$ [s^−1^]		
		AN	ANZ-12	ANZ-48
250	37	6.57 × 10^−9^	6.30 × 10^−9^	1.15 × 10^−8^
275	37	8.21 × 10^−9^	3.70 × 10^−8^	7.22 × 10^−8^
300	26	1.53 × 10^−8^	2.34 × 10^−8^	1.79 × 10^−8^
300	31	1.09 × 10^−8^	4.85 × 10^−8^	1.39 × 10^−7^
300	37	3.79 × 10^−8^	2.28 × 10^−7^	2.00 × 10^−7^
Q_c_-Activation creep energy [kJ]		86.2	178.8	142.9
n-stress exponent		3	6	6

## Data Availability

The original contributions presented in this study are included in the article. Further inquiries can be directed to the corresponding author.
